# Hand-book of Medical Microscopy for Students and General Practitioners

**Published:** 1894-11

**Authors:** 


A Handbook of Medical Microscopy for Students and General Practitioners, Introducing Chapters on Bacteriology, Neoplasms and Urinary Examinations. By James E. Reeves, M. D., Member of the Association of American Physicians; Ex-President of the American Public Health Association; Ex-President of the State Medical Society of West Virginia; Author of "A Practical Treatise on Enteric and Typhoid Fever," etc., etc. With a Glossary and numerous illustrations. Philadelphia. P. Blackiston, Son & Co., 1012 Walnut Street. 1894.

In this day of medicine we must all recognize the importance of the microscope as an aid to diagnosis. Many of our profession recognize this fact, but owing to various circumstances are unable to acquire a knowledge of its use. This book is written by one who mastered the microscope by himself, having for his motto "Where there is a will, there is a way."

Realizing the difficulties which beset the student at every turn in this complicated study, he has sought to make clear all those points which were obstacles to himself, placing in easy grasp through this little book, a thorough knowledge of the microscope for those who are willing to seek knowledge in this way. It is an excellent little book, and will prove of much value to all students of the microscope.

